# Supplementary material for: Unloading of homologous recombination factors is required for restoring double‐stranded DNA at damage repair loci
Source: EMBO J. 2017 Jan 17;36(2):213–31. doi: 10.15252/embj.201694628 (PMC5239998; doi:10.15252/embj.201694628)
Supplement: Supplementary file 1 — Expanded View Figures PDF [file EMBJ-36-213-s001.pdf]

Expanded View Figures

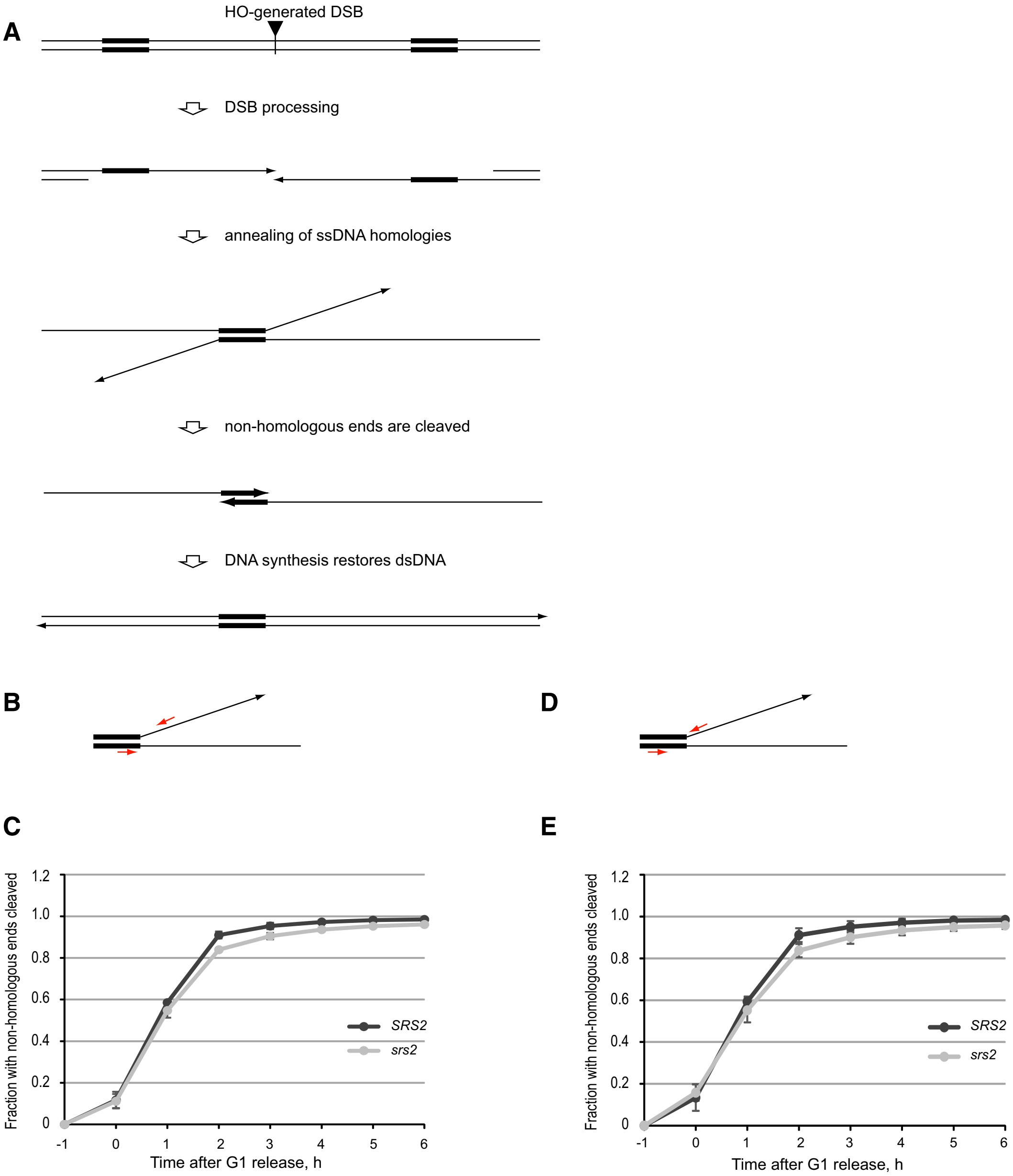

Figure EV1. *srs2*Δ does not affect repair via SSA at the stage of cleavage of non-homologous DNA ends.

A Schematic of DSB repair by SSA. Broken DNA ends are resected to generate long overhangs with 3′-ends (arrows). Once in a single-stranded form, regions of homology (thick lines) are annealed to each other. Non-homologous 3′-ends (thin lines ending with arrows) are cleaved by Rad1/Rad10, and dsDNA is restored via DNA synthesis.

B Schematics of the qPCR-based assays for monitoring the cleavage of non-homologous 3′-DNA ends in the experiments shown in (C). Red arrows indicate qPCR primers.

C Cleavage of non-homologous 3′-ends in *SRS2* and *srs2*Δ cells over a time-course experiment analysed by qPCR using two different sets of primers. Average ± SD (n = 3) is shown for each time point. Strains used: NK4691–4693; NK4805–4808.

D Schematics of the qPCR-based assays for monitoring the cleavage of non-homologous 3′-DNA ends in the experiments shown in (E). Red arrows indicate qPCR primers.

E Cleavage of non-homologous 3′-ends in *SRS2* and *srs2*Δ cells over a time-course experiment analysed by qPCR using two different sets of primers. Average ± SD (n = 3) is shown for each time point. Strains used: NK4691–4693; NK4805–4808.

© 2016 The Authors

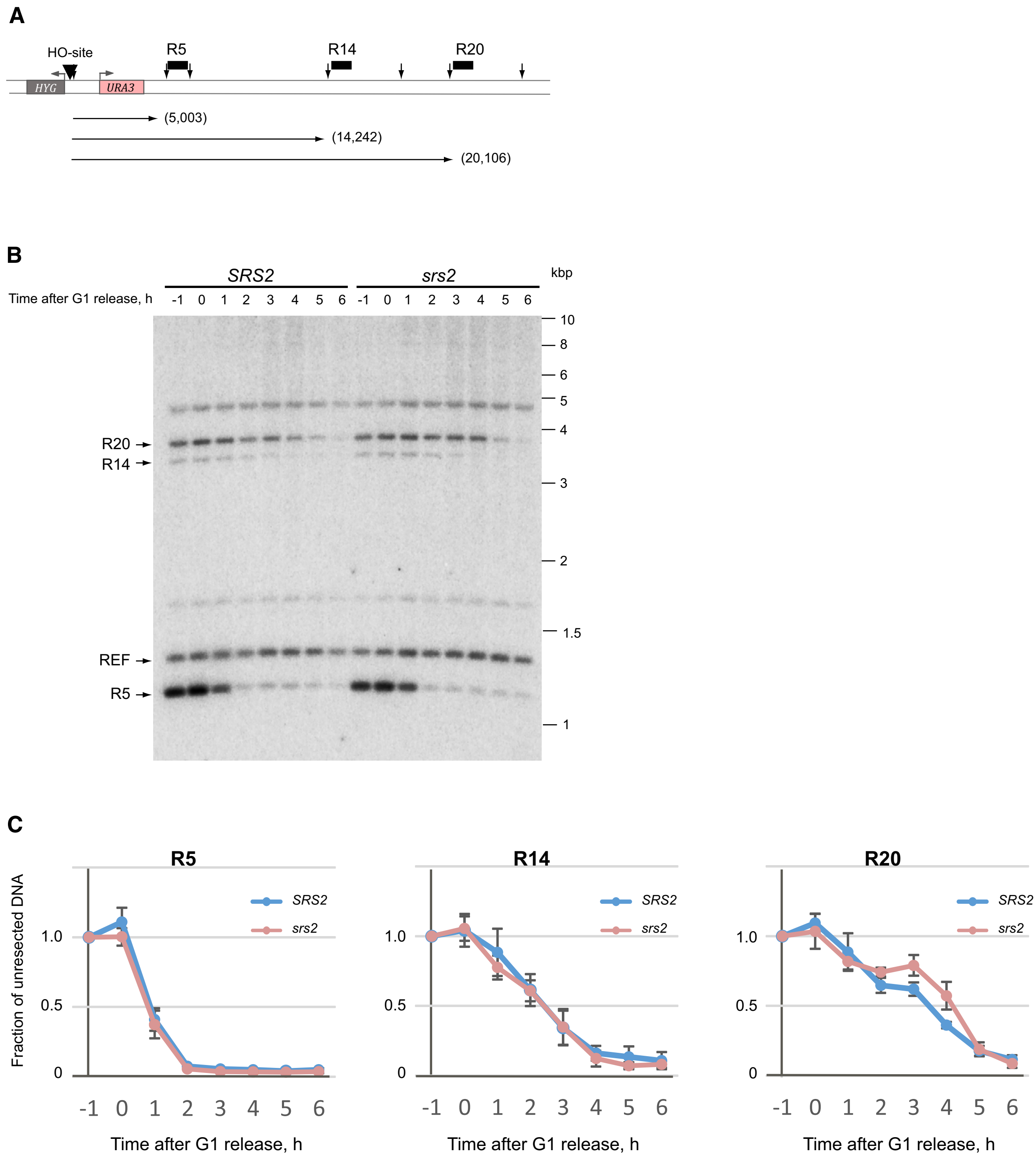

Figure EV2. Loss of Srs2 does not affect DSB resection.

A Schematic of unrepairable DSB used for comparative analysis of break resection in (B and C). HO recognition site is shown by black triangle, vertical arrows represent *Bgl*II restriction sites, and horizontal arrows depict distance (in bp, shown in brackets) from DSB to the three *Bgl*II restriction fragments monitored by Southern hybridization using the corresponding probes (black boxes labelled R5, R14 and R20). Notice, the DNA on the right hand side of the break is identical to that used in SSA assays (Fig 4A), while on the left, *ura3-52–KAN* was replaced by *HYG*, thereby removing homology required for DSB repair by SSA.

B A representative Southern blot image used for quantitative analysis of DSB resection. Four DNA fragments indicated by arrows on the left were used in the analyses: three chr.V fragments affected by resection and a chr.IV fragment containing *ARS1* (*TRP1* locus) used as a normalizer of DNA loading.

C Quantitative analysis of DSB resection in *SRS2* and *srs2* mutant cells over a time-course experiment involving cell synchronization and DSB induction in G1 followed by release into S/G2. Average ± SD (n = 4) is shown for each time point.

Data information: Strains used: NK5728, NK5729; NK5754, NK5755.

© 2016 The Authors

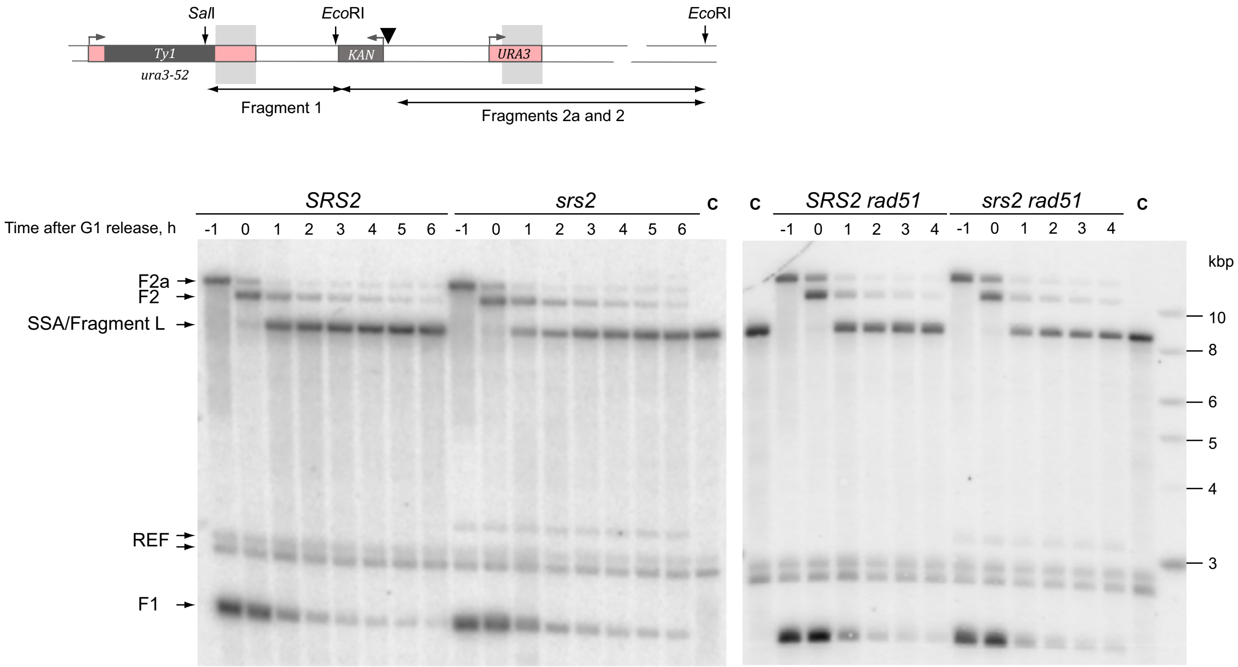

Figure EV3. Analysis of SSA by Southern blot hybridization in *SRS2* and *srs2*Δ cells in the presence and absence of Rad51.

A schematic and representative Southern blot images are shown for fragment L (see Fig 4C for further explanation). A black triangle represents the HO site. Sites for the restriction enzymes used in the analysis of the corresponding DNA fragment are shown as vertical arrows. Hybridization probe spans the region of homology indicated by grey shadows. F1, F2, F2a correspond to fragment 1, 2 and 2a, respectively, indicated by two-ended arrows at the bottom of the schematic; SSA, the product of repair (fragment L); REF, a fragment on chr.IV (*ARS1* locus) detected by the reference probe; C, control strain. Numbers above each gel lane indicate time points in the time-course experiments.

 © 2016 The Authors

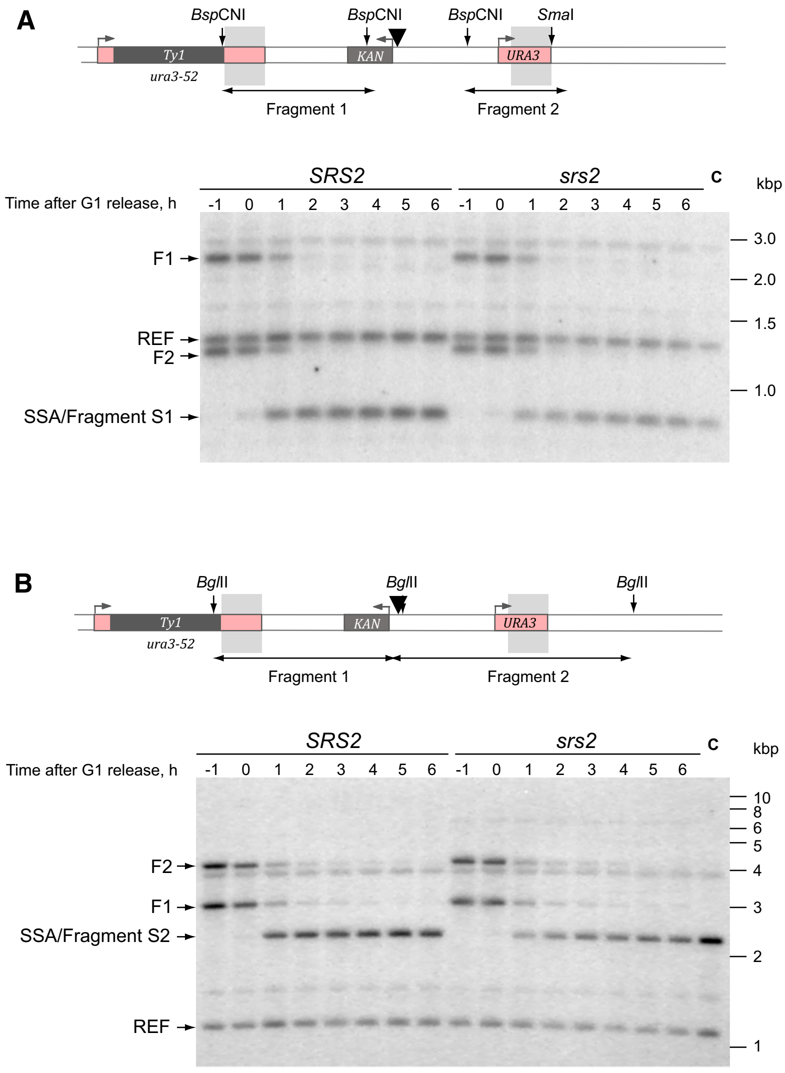

Figure EV4. Analysis of SSA by Southern blot hybridization in *SRS2* and *srs2*Δ cells.

A, B Schematics and a representative Southern blot images are shown for fragment S1 and S2 in panels (A) and (B), respectively (see Fig 5A for further explanation). In each schematic, a black triangle represents the HO site. Sites for the restriction enzymes used in the analysis of the corresponding DNA fragment are shown as vertical arrows. Hybridization probe spans the region of homology indicated by grey shadows. F1 and F2 correspond to fragments 1 and 2, respectively, indicated by two-ended arrows at the bottom of each schematic; SSA, the product of repair (fragments S1 and S2 in panels A and B, respectively); REF, a fragment on chr.IV (*ARS1* locus) detected by the reference probe; C, control strain. Numbers above each gel lane indicate time points in the time-course experiments.

© 2016 The Authors
