# Supplementary material for: Unloading of homologous recombination factors is required for restoring double‐stranded DNA at damage repair loci
Source: EMBO J. 2017 Jan 17;36(2):213–31. doi: 10.15252/embj.201694628 (PMC5239998; doi:10.15252/embj.201694628)
Supplement: Supplementary file 2 — Table EV1 [file EMBJ-36-213-s002.docx]

# Table EV1. *Saccharomyces cerevisiae A364a* strains used in the study

| Strain | Relevant genotype | Construction/Reference |
| --- | --- | --- |
| NK1 | *MATa bar1::LEU2 trp1-289 ura3-5 leu2-3,112* | Makovets & Blackburn, 2009 |
| NK1264 | *MATa-inc trp1-289 ura3::NAT leu2::LEU2-Pgal-HO MNT2::kan::HOsite-URA3-STAR-TEL pif1-m2* | Makovets & Blackburn, 2009 |
| NK1949 | *MATa-inc trp1-289 ura3::NAT leu2::LEU2-Pgal-HO ZRT1::KAN-TG_81_-HOsite-URA3-STAR-TEL* | Multiple steps. Note that there is 2 kb of DNA between the HO site and telomere |
| NK2012 NK2013 | *MATa-inc trp1-289 ura3::NAT leu2::LEU2-Pgal-HO MNT2::kan::HOsite-URA3-STAR-TEL pif1-m2 rad51::TRP1* | NK1264 *rad51::TRP1* |
| NK2014 NK2015 | *MATa-inc trp1-289 ura3::NAT leu2::LEU2-Pgal-HO MNT2::kan::HOsite-URA3-STAR-TEL pif1-m2 rad52::TRP1* | NK1264 *rad52::TRP1* |
| NK2016 NK2017 | *MATa-inc trp1-289 ura3::NAT leu2::LEU2-Pgal-HO MNT2::kan::HOsite-URA3-STAR-TEL pif1-m2 exo1::TRP1* | NK1264 *exo1::TRP1* |
| NK2363 NK2364 | *MATa-inc trp1-289 ura3::NAT leu2::LEU2-Pgal-HO MNT2::kan::HOsite-URA3-STAR-TEL pif1-m2 rad55::HYG* | NK1264 *rad55::TRP1* |
| NK2369 NK2370 | *MATa-inc trp1-289 ura3::NAT leu2::LEU2-Pgal-HO MNT2::kan::HOsite-URA3-STAR-TEL pif1-m2 rad57::HYG* | NK1264 *rad57::TRP1* |
| NK2375 NK2376 | *MATa-inc trp1-289 ura3::NAT leu2::LEU2-Pgal-HO MNT2::kan::HOsite-URA3-STAR-TEL pif1-m2 srs2::HYG* | NK1264 *srs2::HYG* |
| NK2451 NK2452 | *MATa-inc trp1-289 ura3::NAT leu2::LEU2-Pgal-HO MNT2::kan::HOsite-URA3-STAR-TEL pif1-m2 srs2::HYG rad52::TRP1* | NK2375 *rad52::TRP1*  NK2376 *rad52::TRP1* |
| NK2457 NK2458 | *MATa-inc trp1-289 ura3::NAT leu2::LEU2-Pgal-HO MNT2::kan::HOsite-URA3-STAR-TEL pif1-m2 srs2::HYG rad51::TRP1* | NK2375 *rad51::TRP1* |
| NK2469 | *MATa-inc trp1-289 ura3::NAT leu2::LEU2-Pgal-HO MNT2::kan::HOsite-URA3-STAR-TEL pif1-m2 srs2::HYG rad55::TRP1* | NK2375 *rad55::TRP1* |
| NK2473- NK2475 | *MATa-inc trp1-289 ura3::NAT leu2::LEU2-Pgal-HO MNT2::kan::HOsite-URA3-STAR-TEL pif1-m2 srs2::HYG rad57::TRP1* | NK2375 *rad57::TRP1* |
| NK3292 NK3293 | *MATa-inc ura3::NAT leu2::LEU2-Pgal-HO pif1-m2 trp1::HYG MNT2::HOsite-URA3-STAR-TEL* | Multiple steps |
| NK3308- NK3310 | *MATa-inc trp1-289 ura3::NAT leu2::LEU2-Pgal-HO MNT2::kan::HOsite-URA3-STAR-TEL pif1-m2 srs2::srs2(1-860)-TRP1* | NK1264 *srs2::srs2(1-860)-TRP1* |
| NK3332- NK3334 | *MATa-inc trp1-289 ura3::NAT leu2::LEU2-Pgal-HO MNT2::kan::HOsite-URA3-STAR-TEL pif1-m2 srs2::srs2-K41A-TRP1* | NK1264::pYT341/*Msc*I |
| NK3353- NK3355 | *MATa-inc trp1-289 ura3::NAT leu2::LEU2-Pgal-HO MNT2::kan::HOsite-URA3-STAR-TEL pif1-m2 srs2::srs2(1-741)-TRP1* | NK1264 *srs2::srs2(1-741)-TRP1* |
| NK3980 NK3981 | *MATa-inc ura3::NAT leu2::LEU2-Pgal-HO MNT2::KAN-(ARO4 – telomere) HIS7::kan* | Multiple steps |
| NK4070 NK4079 | *MATa-inc ura3::NAT leu2::LEU2-Pgal-HO MNT2::KAN-(ARO4-SPO23)::HOsite-URA3-STAR-TEL HIS7::kan* | Multiple steps. Note that chrVII and II have 6,272 bp of overlapping sequence that provides homology to repair a DSB. |
| NK4112  NK4113 | *MATa-inc ura3::NAT leu2::LEU2-Pgal-HO pif1-m2 trp1::HYG MNT2::HOsite-URA3-STAR-TEL rad51::KAN* | NK3292 *rad51::KAN* |
| NK4114 NK4115 | *MATa-inc ura3::NAT leu2::LEU2-Pgal-HO pif1-m2 trp1::HYG MNT2::HOsite-URA3-STAR-TEL rad52::KAN* | NK3292 *rad52::KAN* |
| NK4217 NK4247 | *MATa-inc trp1-289 ura3::NAT leu2::LEU2-Pgal-HO MNT2::kan::HOsite-URA3-STAR-TEL pif1-m2 srs2::srs2(1-836)-TRP1* | NK1264 *srs2::srs2(1-836)-TRP1* |
| NK4230  NK4231 | *MATa-inc trp1-289 ura3::NAT leu2::LEU2-Pgal-HO ZRT1::KAN-TG_81_-HOsite ADH4::URA3* | Multiple steps. Note that there is 20 kb of DNA between the HO site and telomere |
| NK4232  NK4233 | *MATa-inc ura3::NAT leu2::LEU2-Pgal-HO trp1::HYG MNT2::HOsite-URA3-STAR-TEL pif1-m2:: pif1-m1-TRP1* | NK3293::pYT147/*Bgl*II |
| NK4264  NK4265 | *MATa-inc trp1-289 ura3::NAT leu2::LEU2-Pgal-HO ZRT1::KAN-TG_81_-HOsite ADH4::URA3 srs2::TRP1* | NK4230 *srs2::TRP1*  NK4231 *srs2::TRP1* |
| NK4268  NK4269 | *MATa-inc trp1-289 ura3::NAT leu2::LEU2-Pgal-HO ZRT1::KAN-TG_81_-HOsite-URA3-STAR-TEL srs2::TRP1* | NK1949 *srs2::TRP1* |
| NK4670 NK4671 | *MATa-inc ura3::NAT leu2::LEU2-Pgal-HO pif1-m2 trp1::HYG MNT2::HOsite-URA3-STAR-TEL srs2::TRP1* | NK3292 *srs2::TRP1* |
| NK4691-NK4693 | *MATa-inc trp1-289 leu2::LEU2-Pgal-HO ura3-52::KAN-HOsite-URA3* | Multiple steps |
| NK4805-NK4808 | *MATa-inc trp1-289 leu2::LEU2-Pgal-HO ura3-52::KAN-HOsite-URA3 srs2::TRP1* | NK4691 *srs2::TRP1*  NK4692 *srs2::TRP1* |
| NK5058-NK5061 | *MATa-inc trp1-289 leu2::LEU2-Pgal-HO ura3-52::KAN-HOsite-URA3 srs2::srs2(1-741)-TRP1* | NK4691 *srs2::srs2(1-741)-TRP1*  NK4692 *srs2::srs2(1-741)-TRP1* |
| NK5062-NK5065 | *MATa-inc trp1-289 leu2::LEU2-Pgal-HO ura3-52::KAN-HOsite-URA3 srs2::srs2(1-836)-TRP1* | NK4691 *srs2::srs2(1-836)-TRP1*  NK4692 *srs2::srs2(1-836)-TRP1* |
| NK5066-NK5069 | *MATa-inc trp1-289 leu2::LEU2-Pgal-HO ura3-52::KAN-HOsite-URA3 srs2::srs2(1-860)-TRP1* | NK4691 *srs2::srs2(1-860)-TRP1*  NK4692 *srs2::srs2(1-860)-TRP1* |
| NK5070-NK5073 | *MATa-inc trp1-289 leu2::LEU2-Pgal-HO ura3-52::KAN-HOsite-URA3 exo1::HYG* | NK4691 *exo1::HYG*  NK4692 *exo1::HYG* |
| NK5074-NK5080 | *MATa-inc trp1-289 leu2::LEU2-Pgal-HO ura3-52::KAN-HOsite-URA3 srs2::TRP1 exo1::HYG* | NK4805 *exo1::HYG*  NK4807 *exo1::HYG* |
| NK5081-NK5084 | *MATa-inc trp1-289 leu2::LEU2-Pgal-HO ura3-52::KAN-HOsite-URA3 rad51::HYG* | NK4691 *rad51::HYG*  NK4692 *rad51::HYG* |
| NK5085-NK5091 | *MATa-inc trp1-289 leu2::LEU2-Pgal-HO ura3-52::KAN-HOsite-URA3 srs2::TRP1 rad51::HYG* | NK4805 *rad51::HYG*  NK4807 *rad51::HYG* |
| NK5104-NK5107 | *MATa-inc trp1-289 leu2::LEU2-Pgal-HO ura3-52::KAN-HOsite-URA3 srs2::srs2-K41A-TRP1* | NK4691::pYT341/*Msc*I  NK4692::pYT341/*Msc*I |
| NK5244 NK5245 | *MATa-inc trp1-289 ura3::NAT leu2::LEU2-Pgal-HO MNT2::kan::HOsite-URA3-STAR-TEL pif1-m2 srs2::HYG exo1::TRP1* | NK2375 *exo1::TRP1* |
| NK5321 NK5322 | *MATa-inc ura3::NAT leu2::LEU2-Pgal-HO MNT2::KAN-(ARO4-SPO23)::HOsite -URA3-STAR-TEL HIS7::kan srs2::HYG* | NK4070 *srs2::HYG* |
| NK5446 NK5447 | *MATa-inc ura3::NAT leu2::LEU2-Pgal-HO MNT2::KAN-(ARO4-SPO23)::HOsite-URA3-STAR-TEL HIS7::kan exo1::TRP1* | NK4070 *exo1::TRP1* |
| NK5448 NK5449 | *MATa-inc ura3::NAT leu2::LEU2-Pgal-HO MNT2::KAN-(ARO4-SPO23)::HOsite-URA3-STAR-TEL HIS7::kan srs2::HYG exo1::TRP1* | NK5321 *exo1::TRP1* |
| NK5450 NK5451 | *MATa-inc ura3::NAT leu2::LEU2-Pgal-HO MNT2:: KAN-(ARO4-SPO23)::HOsite-URA3-STAR-TEL HIS7::kan srs2::srs2(1-860)-TRP1* | NK4070 *srs2::srs2(1-860)-TRP1* |
| NK5452  NK5453 | *MATa-inc ura3::NAT leu2::LEU2-Pgal-HO MNT2::KAN-(ARO4-SPO23)::HOsite -URA3-STAR-TEL HIS7::kan srs2::srs2(1-836)-TRP1* | NK4070 *srs2::srs2(1-836)-TRP1* |
| NK5454 NK5455 | *MATa-inc ura3::NAT leu2::LEU2-Pgal-HO MNT2::KAN-(ARO4-SPO23)::HOsite-URA3-STAR-TEL HIS7::kan srs2::srs2(1-741)-TRP1* | NK4070 *srs2::srs2(1-741)-TRP1* |
| NK5536  NK5537 | *MATa-inc ura3::NAT leu2::LEU2-Pgal-HO MNT2::KAN-(ARO4-SPO23)::HOsite-URA3-STAR-TEL HIS7::kan srs2::srs2-K41R-TRP1* | NK4070::pYT341/*Msc*I |
| NK5728 NK5729 | *MATa-inc trp1-289 leu2::LEU2-Pgal-HO HYG-HOsite-URA3* | NK4691 *HYG-HOsite-URA3*  NK4692 *HYG-HOsite-URA3* |
| NK5754 NK5755 | *MATa-inc trp1-289 leu2::LEU2-Pgal-HO HYG-HOsite-URA3 srs2::NAT* | NK5728 *srs2::NAT*  NK5729 *srs2::NAT* |
