# Supplementary material for: Unloading of homologous recombination factors is required for restoring double‐stranded DNA at damage repair loci
Source: EMBO J. 2017 Jan 17;36(2):213–31. doi: 10.15252/embj.201694628 (PMC5239998; doi:10.15252/embj.201694628)
Supplement: Supplementary file 3 — Table EV2 [file EMBJ-36-213-s003.docx]

# Table EV2. Oligonucleotides used in the study

| Oligonucleotide | Sequence | Purpose |
| --- | --- | --- |
| OSM189 | 5’-ACGCCAGAAAATGTTGGTGATGCGCTT-3’ | *ARS*1 probe |
| OSM190 | 5’-ATCCACATCAATGGCTAATGGCAAAACT-3’ |  |
| OSM937 | 5’-AGCAGGATATTCAGCGGTGT-3’ | RS2.6 probe |
| OSM1006 | 5’-TGACTGGTACTACCGTAACGGTTC-3’ | qPCR at the *ARO1* locus |
| OSM1007 | 5’-GAATACCATCTGGTAATTCTGTAGTTTTGAC-3’ |  |
| OSM1370 | 5’-cccccggccgAAGATACATAGGGTCAGGCAT-3’ | pYT341 construction |
| OSM1371 | 5’-ccccgtcgacTTCGAATCTAAGATGTATTC-3’ |  |
| OSM1372 | 5’-GTCCCGGCACAGGcgccACTAAGGTTTTAA-3’ |  |
| OSM1373 | 5’-TTAAAACCTTAGTggcgCCTGTGCCGGGAC-3’ |  |
| OSM1487 | 5’-CCACACACACCACACCCACACACCACACCC-3’ | *de novo* telomere addition qPCR |
| OSM1502 | 5’- CAAGGAAGGTGAGTTTGGAAAGTTGATC-3’ |  |
| OSM2161 | 5’-TGTGGATATCTTGACTGATTTTTCC-3’ | *URA3* probe |
| OSM2162 | 5’-ATACATGCATTTACTTATAATACAG-3’ |  |
| OSM2233 | 5’-TGTATACTAAACTCACAAATTAGAGC-3’ | qPCR detection of long non-homologous 3’-ends after SSA |
| OSM2234 | 5’-CAACACTCAACCCTATCTCG-3’ |  |
| OSM2242 | 5’-AGGGATGCTAAGGTAGAGG-3’ | qPCR detection of short non-homologous 3’-ends after SSA |
| OSM2244 | 5’-CTCCTTACGCATCTGTGC-3’ |  |
| OSM2287 | 5’-GATGGTAGTCAATAAGCAGTCC-3’ | qPCR at the *UBC5* locus |
| OSM2288 | 5’-TTTGAAGCATGGCTGTGG-3’ |  |
| OSM2332 | 5’-AGGAGAAAGTATCAGTACATTGC-3’ | RS6.8 probe |
| OSM2333 | 5’-GGAACTTTCATAGTAGATTAGCC-3’ |  |
| OSM2336 | 5’-GTTACGGTGAAAGTTATGAGAGC-3’ | BIR6 probe |
| OSM2337 | 5’-GAAGTGATCTCTCCATCTGCTGC-3’ |  |
| OSM2338 | 5’-GATTTGCCATCGCCAGATGAAGG-3’ | BIR36 probe |
| OSM2339 | 5’-TGTTCCAGGCTGTTGAAGTAGCC-3’ |  |
| OSM2340 | 5’-TGGTATTCCACACCAAAGCGAGG-3’ | BIR77 probe |
| OSM2341 | 5’-AAGTCCACTGATGATATCCCACC-3’ |  |
| OSM2344 | 5’-GCATTATTTGCCTCGGCAGATGG-3’ | ARS522 probe |
| OSM2345 | 5’-CGAAATTGAAGCTCGCGCTAACC-3’ |  |
| OSM2347 | 5’-TTATCTCTGATATTACCACCTGG-3’ | RS15.2 probe |
| OSM2348 | 5’-GTCTATGTCAGATTATCTATTCC-3’ |  |
| OSM2380 | 5’-TAGAACCAGTTCAAAGTAGCAGC-3’ | RS2.6 probe |
